# Editorial: HIV and the gut: novel insights into HIV pathogenesis, clinical implications and therapeutic approaches

**DOI:** 10.3389/fimmu.2026.1809780

**Published:** 2026-03-12

**Authors:** Sonia Moretti, Gabriella d’Ettorre, Ivan Schietroma, Claudia Matteucci, Wondwossen Degu

**Affiliations:** 1 National Institute of Health (ISS), Rome, Italy; 2 Department of Public Health and Infectious Diseases, Faculty of Pharmacy and Medicine, Sapienza University of Rome, Rome, Italy; 3 Department of Experimental Medicine, University of Rome Tor Vergata, Rome, Italy; 4 Addis Ababa University, Addis Ababa, Ethiopia

**Keywords:** comorbidities, dysbiosis, GALT, gut microbiota, HIV/AIDS, HIV-1 reservoir, immune activation, probiotics/prebiotics

The gastrointestinal tract is increasingly recognized as a central player in HIV pathogenesis. From the earliest stages of infection, HIV profoundly disrupts the intestinal epithelial barrier and gut-associated lymphoid tissue (GALT), leading to microbial dysbiosis and translocation, and persistent immune activation, despite effective antiretroviral therapy (ART) (1, 2). Severe depletion of CD4+ T cells, particularly Th17 cells, together with an altered Treg/Th17 balance, compromises mucosal integrity and sustains chronic inflammation, while the gut remains a major reservoir for HIV (3, 4). Collectively, these alterations contribute not only to viral persistence but also to the development of multiple HIV-associated comorbidities.

The original research articles and reviews, collected in this Research Topic, provide new insights into the immunological, metabolic and microbiota-driven mechanisms linking HIV infection to gut dysfunction.

A major barrier to HIV cure is the persistence of long-lived latent reservoirs that evade immune clearance and ART. Among all tissues, the gastrointestinal tract, particularly GALT, harbors the majority of infected cells even under suppressive ART. Lau et al. present the gut not as a passive reservoir but as an active driver of HIV latency, immune activation, and reservoir maintenance during long-term ART. Early in infection, HIV induces rapid and profound depletion of CCR5^+^ memory CD4^+^ T cells, including Th17 and Th22 subsets that are critical for maintaining epithelial integrity. This damage initiates a cycle of barrier dysfunction, microbial translocation, and inflammation that persists despite ART. Rather than resolving, persistent immune activation sustains viral reservoirs and continuously generates new HIV-susceptible target cells. The authors highlight gut-enriched cellular reservoirs, including CCR6^+^ CD4^+^ T cells and tissue-resident memory T cells, which exhibit deep latency, long lifespan, and limited accessibility to immune-mediated clearance. Although multiple strategies have been explored, ranging from latency-reversing agents and immune-based therapies to microbiome modulation and trafficking inhibitors, none have achieved so far durable reduction of gut reservoirs.

Expanding on the role of the epithelial compartment, Creighton et al. highlight specialized intestinal epithelial cells as active participants in shaping the gut reservoir. Microfold (M) cells and interferon-stimulated gene (ISG)-expressing enterocytes display high expression of ISGs such as ISG15 and maintain close interactions with immune cells. While interferon responses are classically antiviral, chronic or dysregulated interferon signaling in HIV infection may paradoxically promote immune activation, T-cell proliferation, and viral reactivation, thereby reinforcing reservoir persistence. ISG15 exemplifies this dual role, combining intracellular antiviral activity with extracellular cytokine-like functions that may stimulate inflammatory pathways and latent provirus reactivation. Importantly, elevated ISG expression in enterocytes can occur independently of canonical interferon signaling, implicating microbial sensing, cellular stress, and chronic inflammation. These observations underscore how the local gut microenvironment may influence the efficacy of both “shock and kill” and “block and lock” cure strategies.

Despite effective viral suppression, a subset of people with HIV, termed immunological non-responders (INR), fail to adequately restore CD4^+^ T-cell counts. Kaarbø et al. provide important insight by identifying region-specific gut mucosal failure as a hallmark of immune non-response. By integrating transcriptomic and proteomic analyses of gut biopsies, the study demonstrates that, while the terminal ileum remains relatively preserved, the sigmoid colon of INR individuals exhibits profound molecular dysregulation. Moreover, the authors describe selective impairment of B cell-mediated immunity in the sigmoid colon, characterized by defective plasmablast differentiation and reduced immunoglobulin-related pathways. Proteomic data further reveal increased expression of stress- and apoptosis-related proteins alongside reduced abundance of factors involved in epithelial repair and antibody production. The convergence of transcriptomic and proteomic alterations defines a robust molecular signature distinguishing INR from immune responders, providing potential biomarkers and targets for therapeutic intervention.

Frailty has emerged as a major and underrecognized complication of aging with HIV. Xu et al. identify intestinal barrier dysfunction as a key biological correlate of frailty in aging PWH. A central finding was the identification of intestinal fatty acid–binding protein (I-FABP) as an independent biomarker of frailty. Elevated I-FABP levels distinguished frail and pre-frail individuals from non-frail controls and increased progressively with frailty severity, independent of HIV disease markers and psychosocial factors. This suggests that epithelial injury may actively contribute to the biological cascade leading to frailty. The independent association of interferon-induced protein 10 (IP-10) further underscores the role of sustained immune activation. Together, these findings highlight the gut-immune axis as a potential driver of frailty and point toward interventions aimed at preserving barrier integrity and reducing inflammation to promote healthy aging in PWH.

Mitochondrial dysfunction is increasingly recognized as a contributor to HIV- and ART-associated age-related comorbidities. Ma et al. discussed the central role of mitochondrial DNA (mtDNA) damage in HIV-associated inflammaging and immunometabolic disease. Both HIV infection and long-term ART converge on mtDNA integrity, leading to impaired oxidative phosphorylation, metabolic exhaustion of immune cells, and chronic inflammatory signaling that persists despite viral suppression. The authors highlight mtDNA integrity as a potential biomarker for ART toxicity, immune recovery, and disease progression, while also discussing emerging therapeutic strategies aimed at protecting or editing mtDNA, although clinical translation remains limited.

Gut microbiota dysbiosis, characterized by reduced diversity and altered composition, has been independently associated with both HIV infection and cancer. Xie et al. characterized the gut microbiota of PWH with Kaposi’s sarcoma, lymphoma, lung cancer, or colorectal cancer and compared them with HIV-positive individuals without cancer and HIV-negative controls. Using 16S rRNA gene sequencing, the study identified microbial signatures associated with HIV-associated cancers. While HIV infection alone reduces microbial diversity, the presence of cancer markedly exacerbates these alterations, resulting in the lowest alpha diversity and most distinct microbial profiles across cancer types. Associations between depleted commensal bacteria, enrichment of pathogenic taxa, and cancer-related metabolic pathways support a model in which gut microbiota disruption contributes to immune dysregulation and tumor development in HIV.

The gut-brain axis has gained increasing attention as a mediator of immune activation, metabolic homeostasis, and neurocognitive outcomes. Borgognone et al. explored the gut-brain axis using integrated metagenomic and metabolomic analyses in early-treated people with HIV stratified by cognitive performance. Specific microbial species and metabolic pathways, particularly those related to sulfur metabolism and lipid dysregulation, associated with lower cognitive scores. Integrated microbial-metabolomic indices correlated with cognitive performance, functional status, and quality of life, supporting their potential as biomarkers of neurocognitive vulnerability.

Finally, Bai et al. provide exploratory evidence linking the gut microbiome to pulmonary comorbidities through the gut-lung axis. While overall microbial diversity did not differ between individuals with rapid versus stable lung function decline, specific compositional shifts were identified. A distinct set of opportunistic or potentially pathogenic taxa was enriched in individuals with accelerated decline, and a microbiome-derived dysbiosis index was independently associated with lung function decline and airflow limitation, highlighting the potential role of gut microbial composition in pulmonary disease in HIV.

In summary, the contributions to this Research Topic underscore the multifaceted role of the gut microbiome in HIV disease progression and persistence, highlighting the gut as a critical target for future therapeutic strategies aimed at improving immune recovery, reducing comorbidities, and advancing HIV cure efforts.

**Figure 1:**
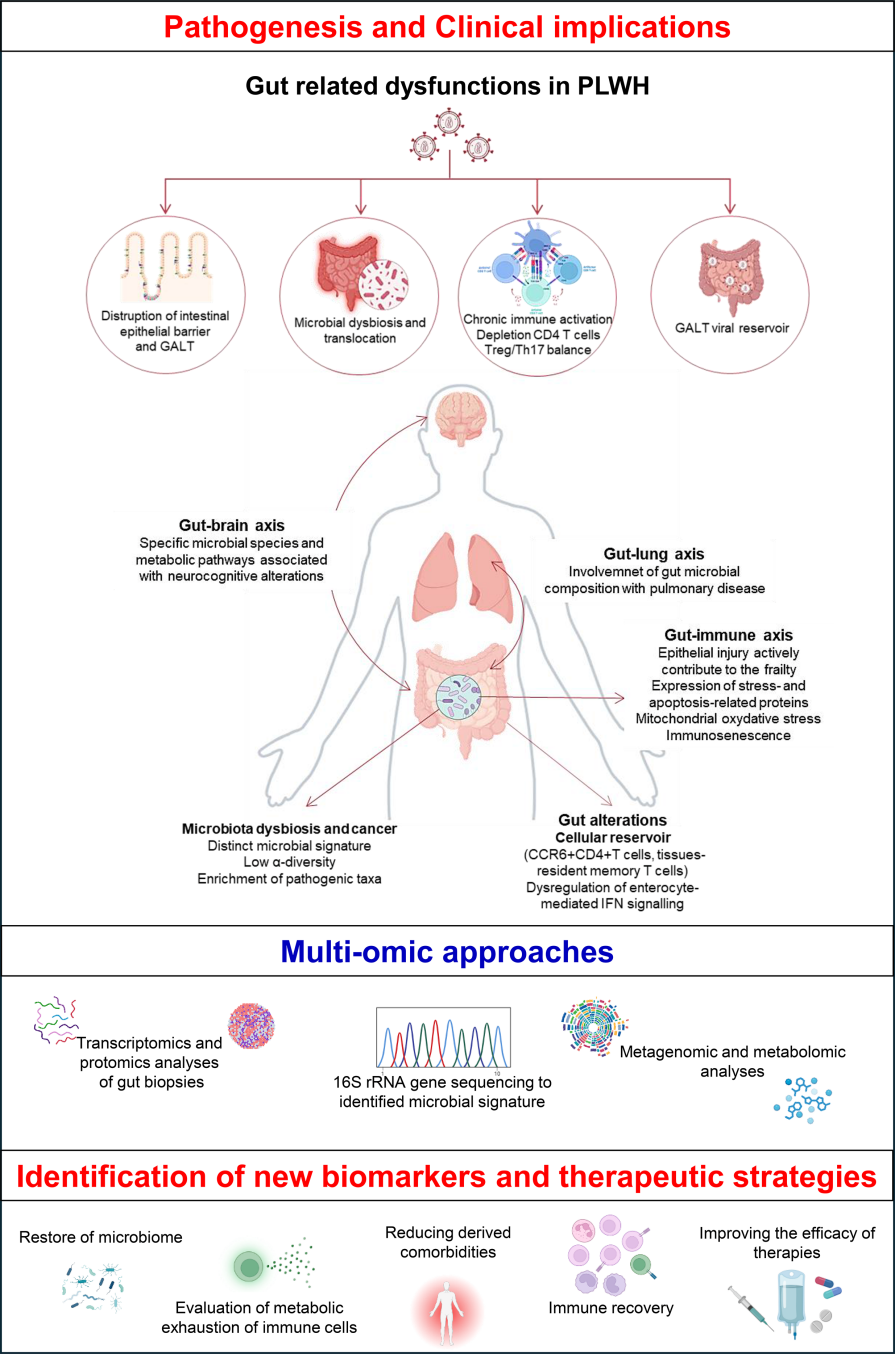
*Gut dysfunction in people living with HIV (PLWH): pathogenesis, clinical implications and therapeutic perspectives.* HIV-associated gut barrier disruption and microbial dysbiosis contribute to immune activation, metabolic exhaustion of immune cells, and HIV-related comorbidities. Integrated multi-omic approaches enable identification of microbial signatures, host pathways, and candidate biomarkers. These strategies support development of targeted interventions aimed at microbiome restoration, immune recovery, reduction of secondary comorbidities, and optimization of therapeutic efficacy. Figure created with BioRender.com, and optimized by PowerPoint.
